# Acute Effect of Quadriceps Myofascial Tissue Rolling Using A Mechanical Self-Myofascial Release Roller-Massager on Performance and Recovery in Young Elite Speed Skaters

**DOI:** 10.3390/sports7120246

**Published:** 2019-12-07

**Authors:** Shaher A. I. Shalfawi, Eystein Enoksen, Håvard Myklebust

**Affiliations:** 1 Department of Education and Sports Science, University of Stavanger, 4021 Stavanger, Norway; havard.myklebust@uis.no; 2 Department of Physical performance, Norwegian School of Sport Sciences, 0806 Oslo, Norway; eystein.enoksen@nih.no

**Keywords:** VO_2peak_, blood lactate, Wingate

## Abstract

Objectives: The main purpose of the present study was to investigate the acute effects of myofascial tissue rolling on endurance performance and recovery using a novel designed mechanical self-induced multi-bar roller-massager. Methods: a randomized crossover, repeated measure design was used. Eight national levelled, junior and neo-senior, speed skaters underwent a 10 min myofascial quadriceps rolling pre- and fifteen minutes post- a stepwise incremental cycling-test to exhaustion followed by a Wingate performance-test. The myofascial quadriceps rolling was used in one out of two laboratory testing-days. Time to exhaustion, peak oxygen uptake (VO_2peak_), blood lactate concentration during 30 min of recovery, and peak- and mean- power during the consecutive Wingate test were recorded. Results: Myofascial quadriceps rolling using roller-massager resulted in higher blood lactate concentration at exhaustion and a larger blood lactate clearance after 10 min to post exhaustion test (both *p* < 0.05), a tendency for a positive effect on Wingate peak-power (*p* = 0.084; *d* = 0.71), whereas no marked differences were observed on VO_2peak_, time to exhaustion and Wingate mean-power. Conclusion: Despite indications for potential benefits of the quadriceps myofascial tissue release using the mechanical self-induced multi-bar roller-massager on blood lactate concentration and Wingate peak-power, the myofascial tissue release gave no marked performance improvements nor indications of negative effects. Future studies could examine the long-term effects of myofascial tissue release on performance and recovery. Furthermore, integrating a measure of the participants’ subjective experience pre- and post the myofascial tissue release would be of great interest.

## 1. Introduction

It is well documented that recovery between exercises is one of the limiting factors for athletic performance particularly between consecutive exercises with limited recovery time [1,2,3]. This could be explained by the amount of adenosine triphosphate (ATP) that is stored in the human body (at any given time), which has been reported to be between ~80–100 g; a reduction in ATP stores triggers the phosphagen system to maintain the concentration of ATP by activating creatine kinase reaction [4]. During continuous high intensity exercise, the breakdown of carbohydrates to resynthesize ATP occurs through a process called glycolysis [4,5]. Pyruvate is the result of glycolysis and it converts into lactate when ATP resynthesis occurs at a very fast rate [4,5]. Therefore, blood lactate measures have been used as a recovery marker in training and between competitions [6]. Research indicates that measuring lactate clearance between successive high intensity competitions that depends on glucose as the primary source of ATP resynthesis could be an indication of the athlete readiness for the next event [3,7]. The importance of recovery can be illustrated by the observed reduction (~6%) in mean power output after 6 min of recovery between two 30 s of sprints [1], the reduced cycling performance with 20 min recovery between two 5 km (~380 s) time trials [3], the observed decrease in accumulated oxygen deficit between two time trials (~205 s) on a roller-skiing treadmill separated by 25 min recovery [2], and the reduced running performance with 30 min of recovery between two 800 m (~145 s) time trials [7]. Hence, speed of recovery could be seen to be more crucial when recovery time is limited.

From this perspective, several modalities have been introduced to speed up recovery between training and competition sessions, such as massage [8], active recovery [9], cryotherapy [10], contrast temperature water immersion [11], hyperbaric oxygen therapy [12], nonsteroidal anti-inflammatory drugs [13], compression garments [14], stretching [15], and foam rolling [16,17]. Due to its practicality, athletes’ self-myofascial release using rollers for the purposes of rehabilitation and enhancing mobility [18], as a substitute for massage, has been widely observed in the past few years. Among others, several studies have examined the effect of self-myofascial release on joint range of motion [19,20], recovery from exercise-induced muscle damage [16], recovery between high intensity bouts [7,21], recovery after competition [17], and performance if applied pre-competition [22]. The main differences between the reported self-myofascial release tools can be summarized in two stances: (i) applied pressure and (ii) rolling cadence. E.g., using tennis balls and foam rollers, the applied pressure on the myofascial tissue is determined by the force exerted on the object using partial body mass or body mass and the rolling cadence is controlled by the person [7,23]. Using the self-administrated roller massage-stick, the subject applies force and controls the rolling cadence on the myofascial tissue [24]. In contrast, a consistent pressure (i.e., force applied on the myofascial tissue using weight plates) combined with a fixed rolling cadence has been reported for the mechanical-roller massage bar [20,25]. Furthermore, the effect of the mechanical-roller massage bar on joint range of motion, maximum voluntary contraction, electromyography activity during exercise and pain has been explored [20,25]. However, to date, the effect of the mechanical self-induced (where athletes using their body mass to apply force on a fixed rolling cadence) multi-bar myofascial tissue roller-massager on recovery and performance has yet to be investigated.

An example of a sport with a limited recovery time is the traditional endurance sport of speed skating. Skaters often race twice a day, and they can only claim 30 min rest between races (International Skating Union, rule 246) [26]. Furthermore, speed skating is characterized by a low quasi-static posture including small knee angles (to reduce the aerodynamic drag), which is needed to achieve a high speed. This posture limits the blood flow to the quadriceps muscles [27] and reduces the VO_2peak_ while skating [28], which makes anaerobic capacity crucial for speed skating performance [29]. E.g., the prestigious 1500 m distance (world record of 101.02 and 110.85 s for men and women, respectively) require about 55% aerobic and 45% anaerobic energy from the speed skaters [30]. Further, skaters use lots of cycling in their training making the testing of their aerobic power and anaerobic capacity to be conducted normally under controlled conditions using Bicycle Ergometer. Such tests have been found to reflect the 1500 m performance changes on the ice at an individual level [29]. Therefore, the purpose of the present study was to investigate the acute effect of quadriceps myofascial tissue release using a mechanical self-myofascial release roller-massager on performance and recovery in young elite speed skaters. We hypothesized that quadriceps myofascial tissue release could potentially affect aerobic and anaerobic performance through reducing locally limitations of aerobic power and/or anaerobic capacity.

## 2. Materials and Methods

### 2.1. Study Design and Participants

**Figure 1:**
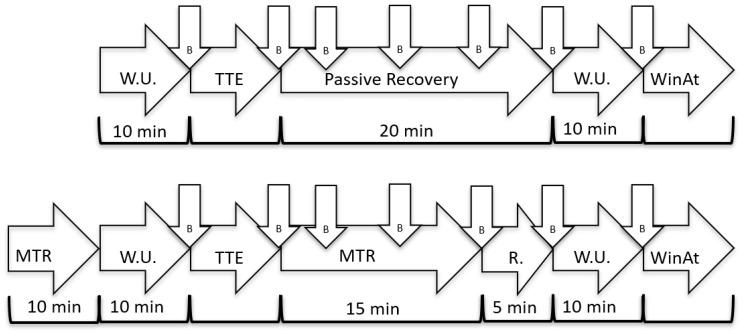
Testing procedure with (upper figure) and without (lower figure) quadriceps myofascial tissue release. MTR = myofascial tissue release; B = blood lactate concentration measure; W.U. = warm up; TTE = test to exhaustion; R. = rest; and WinAt = Wingate anaerobic test.

A random crossover, repeated measure design was implemented to investigate the effects of a novel designed mechanical self-induced multi-bar roller-massager on athletes’ recovery and performance. In a randomized order, participants were provided with a 10 min quadriceps myofascial tissue release pre and 15 min post a cycling test to exhaustion in one of two testing days where the second day was a control day. Peak oxygen uptake (VO_2peak_), respiratory exchange ratio (RER), blood lactate concentration and measures of performance were collected throughout the testing procedure (Figure 1).

### 2.2. Participants

Eight local junior and neo-senior speed skaters aged 18.6 ± 1.3 years (mean ± standard deviation (SD)), body mass 63.8 ± 6.6 kg, and height 171.5 ± 7.9 cm, volunteered to participate in the present study. To be included in the study, the participants had to be ≥16 years old, share the same training routines, be qualified for the Norwegian championships, have no sleep or nutrition irregularity, and perform a restitution training on the day before both days of testing. The included skaters consisted of 4 males (age: 18.5 ± 1.9 years, body mass: 67.9 ± 4.7 kg, height: 178.5 ± 2.7 cm) and 4 females (age: 18.8 ± 0.5 years, body mass: 59.7 ± 6.1 kg, height: 164.5 ± 1.6 cm). In addition to ice- and roller-skating, a normal training year for the participants included cycling, strength and power training, and different dry land exercises in skating positions without skates. In addition to 3–6 individual training sessions per week, the participants trained together with 4–6 sessions per week from May until mid-March. According to their coaches’ training plan, athletes are required to do a total of 685 h of training and racing per year. All participants were healthy at the time of testing with none reporting any ongoing injuries affecting their cycling performance. Written informed consent was obtained from all subjects after a verbal and written explanation of the experimental design and potential risks associated with participating in the study. The study was conducted according to the Helsinki Declaration and the Norwegian National Committees for Research Ethics. This study is registered at the Norwegian Centre for Research Data (id: 58950/3/LT) and the current research information system in Norway under project id: 568684.

### 2.3. Procedure

One month prior to testing, participants performed a stepwise incremental lactate threshold test to determine the participants heart rate (HR) and external load representing 4 mmol/l of blood lactate concentration. These results determined the workload at warm-up and at the first stage of the test to exhaustion during the official testing days. The test was conducted on a stationary Stage SC3 bike (Stages Cycling, Boulder, Colorado, USA), using a fixed (90 revolutions per minute (rpm)) cadence. The test leader registered workload (watts), HR, rate of perceived exertion [31], and blood lactate concentration every fifth minute and before the workload was manually increased by 25 watts. The test ended when the blood lactate concentration reached ≥ 4 mmol/L.

#### 2.3.1. Anthropometric Measurements and Warm-Up

On the first testing day, participants’ height and mass were recorded using a wall mounted Seca stadiometer model 222 and Seca flat digital scale model 877, respectively (Seca Medical Measuring Systems and Scales, Hamburg, Germany). Then participants conducted a warm-up consisting of 10 min cycling on an indoor Star Trac spinning bike (Star Trac Spinner, model: NXT 7090; Irvin, CA, USA). The first 5 and last 3 min of the warm-up were performed at HR equal to 40 beats per minute (BPM) subtracted from HR at lactate threshold. In between, 2 times 45 s with 15 s of recovery was conducted on a HR equal to the lactate threshold. After the warm-up was completed the participants’ blood lactate concentration was measured using Lactate Pro2 (model: LT-1730; Arkray Factory Inc., Kyoto, Japan) (Figure 1).

#### 2.3.2. Test to Exhaustion

After warm-up, the participants adjusted the seat and handlebar of the electro-magnetic test-cycle ergometer (Lode Excalibur, Model: sport 925900; Lode BV, Groningen, Netherlands) to fit their individual versatile position. Prior to test start, mask size was chosen to insure headspace correction and the Vyntus CPX gas analyzer (Vyaire medical; model: versatile JAEGER; Hoechberg, Germany) was calibrated using the fully automated 2-point gas calibration of O_2_/CO_2_, through a special twin tube sample line combined with a fresh air flush system [32]. Then, the test to exhaustion started using a continuous incremental test starting at 125 W for female and 200 W for male athletes and increased by 25 W/min until exhaustion. Participants were instructed to maintain a pedaling rate of 90 rpm [33,34] and the test was stopped if the rpm fell below 80 or when the participant could no longer continue. VO_2peak_ and RER were measured using the breath-by-breath method powered by Sentry Suite software version 2.21 (Vyaire medical; Hoechberg, Germany). The following criteria had to be met for the measures to be accepted: (i) VO_2_ plateaued despite increased exercise intensity; (ii) RER >1.1; and (iii) a post-exercise blood lactate concentration >9 mmol·L^−1^ [34,35]. Time to exhaustion and blood lactate concentration were registered once the test was completed, and blood lactate concentration was further measured at 5, 10, 15, and 20 min (Figure 1).

#### 2.3.3. Intervention

**Figure 2:**
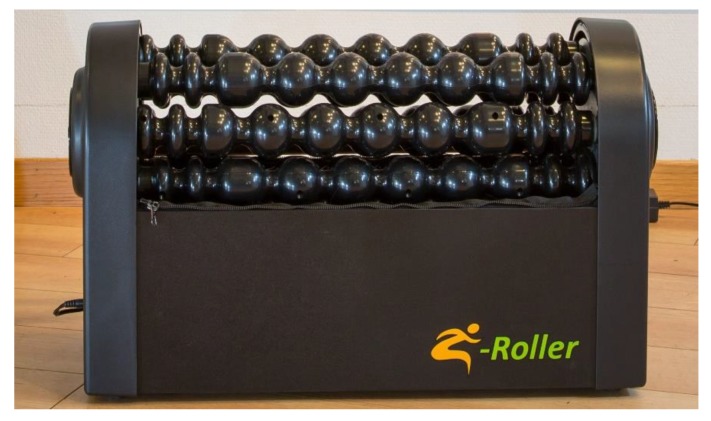
Z-Roller mechanical self-induced multi-bar roller-massager.

**Figure 3:**
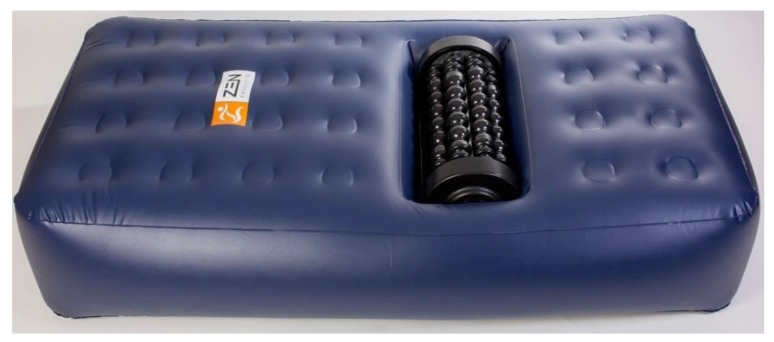
The Z-mattress placed over the Z-Roller.

**Figure 4:**
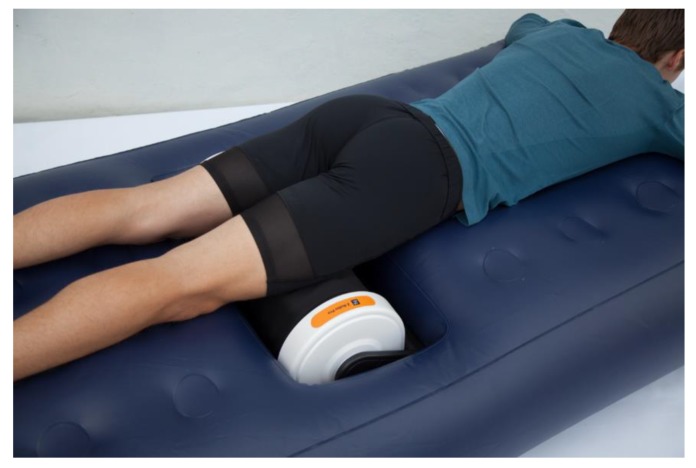
Participant laid face down on the mattress with the quadriceps muscle group placed on the Z-Roller.

To measure the acute effect of pre-performance myofascial tissue release on test to exhaustion performance, participants started one of the testing days with a 10 min [36,37] quadriceps myofascial tissue release using the Z-Roller mechanical self-induced multi-bar roller-massager (Model: V.802; Zen Products, Jessheim, Norway) with a rolling speed of 7.36 m/min. According to the manufacturer, the Z-Roller (Figure 2) intends to give both transverse and circular massages simultaneously, which gives a feeling closer to a regular massage. However, to be able to release the quadriceps myofascial tissue, the Z-mattress (Zen Products, Jessheim, Norway) was placed over the Z-Roller (Figure 3). The Z-mattress has an integrated air pump that allows participant to adapt the air pressure by lowering or raising the mattress, which permits the increase or decrease of their body mass pressure on the Z-roller. The participants laid face down on the mattress with the quadriceps muscle group placed on the Z-Roller (Figure 4). Furthermore, to measure the effect of post-performance myofascial tissue release (between competition recovery), participants started with a 15 min [3,38,39] quadriceps myofascial tissue release using the Z-Roller after the test to exhaustion was completed. All measures were identical to the measures collected on the control test day, including time intervals (Figure 1).

#### 2.3.4. Wingate Anaerobic Cycle Ergometer Test

The Wingate anaerobic 30 s cycling all out test has been shown to be a strong predictor for 1500 m speed skating performance, even for elite athletes at an individual level [29]. Further, to simulate the allowed minimum recovery (according to ISU-rule number 246) [26], the participants had only 30 min between “test to exhaustion” and the “Wingate anaerobic test”. Participants were requested to perform a second 10 min warm up (as described earlier) prior to the 30 s Wingate test performance followed immediately with measuring blood lactate concentration (Figure 1). To eliminate the generated power output before the test, the 30 s Wingate all out test was started from a stationary position with feet fixed to the pedals [29,40]. When the test leader gave the start signal, the athlete started pedalling with maximal effort for 30 s against a constant braking torque of 0.8 Nm/kg for male and 0.77 Nm/kg for female [40,41]. Pedal rate was registered for each second and peak power (Watt), relative peak power (watt/kg), average power (Watt), and rate of fatigue percentage were calculated using a combination of mean pedal rate and the product of braking torque.

### 2.4. Statistical Analysis

Data were transferred to a PC running Microsoft Windows 10 for further analyses. First, the Shapiro–Wilk normality test was performed using GraphPad Prism version 6.00 for Windows (GraphPad Software, La Jolla, CA, USA) on all the measured variables resulting in normally distributed data. Then, 2-way mixed intraclass correlation (ICC) reliability was performed using IBM SPSS Statistics for Windows, version 25 (IBM Corp., Armonk, NY, USA) on all measured variables, and the results showed an ICC of >0.93 with *p* < 0.01. To assess differences in central tendencies (mean) between the results from the day with myofascial tissue release and the results from the day without myofascial tissue release, paired *t*-test was performed on each measured variable using GraphPad Prism. For a better understanding of the differences [42], results were presented as mean difference, standard error of the difference, 95% confidence interval (95% CI) with the effect size (Cohen *d*) calculated and defined as small when *d* = 0.2–0.49, medium when *d* = 0.5–0.79 and large when *d* ≥ 0.8 [43]. The alpha level for statistical significance was set to *p* ≤ 0.05 for all statistical examinations.

## 3. Results

### 3.1. The Effect of Pre Exercise Myofascial Tissue Release on Performance to Exhaustion

All participants in this study met the criteria set for the cycle test to exhaustion, including plateaued VO_2_, RER > 1.1 and a post-exercise blood lactate concentration > 9 mmol·L^−1^ (Table 1). The results further showed that a 10 min pre warm-up myofascial tissue rolling had a trivial effect on VO_2Peak_ (*p* = 0.65; *d* < 0.2), and small effect on time to exhaustion (*p* = 0.555; *d* < 0.49) and RER (*p* = 0.462; *d* < 0.49; Table 1). Furthermore, blood lactate concentration was statistically significantly higher (*p* < 0.05) after test to exhaustion on the day with myofascial tissue release with a large effect size (*d* > 0.8; Table 1).

### 3.2. The Effect of Myofascial Tissue Release on Blood Lactate Concentration between High Intensity Exercises

During the 20 min post exercise to exhaustion, blood lactate clearance was on average higher at each testing time-point when using 15 min of post exercise myofascial tissue release compared to no release (Table 2). However, only the accumulated drop in blood lactate concentration at 10 min post exhaustion test was statistically different with a large effect size (*p* = 0.004; *d* > 0.8). Further, the rate of blood lactate clearance was on average 0.39 mmol·L^−1^·min^−1^, with no statistically significant differences between testing time-points, nor between testing conditions (Table 2).

### 3.3. The Effect of Myofascial Tissue Release on the Consecutive High Intensity Exercise

None of the measured variables from the Wingate anaerobic cycle ergometer test had a statistically significant difference (Table 3). However, the average results indicated a tendency for a moderately higher (*d* = 0.7) peak power output on the day with myofascial tissue rolling compared to the day without myofascial tissue rolling (*p* = 0.084). Further, the results indicate a small effect on rate of fatigue (*d* < 0.49), and a trivial effect on 30 s average power (*d* < 0.2).

## 4. Discussion

The present study was designed to investigate the acute effect of quadriceps myofascial tissue rolling on performance and recovery using a novel designed mechanical self-induced multi-bar myofascial tissue roller-massager on 8 young elite speed skaters. The main findings of 10 min pre-warm-up myofascial tissue rolling were; (i) a statistically significantly higher blood lactate concentration at the end of the exercise to exhaustion test (1.75 ± 2.06 mmol·L^−1^; *p* < 0.05; *d* > 0.8) and; (ii) no statistical significance combined with trivial and small effects on VO_2peak_ and time to exhaustion, respectively. Further, the main finding during recovery after the exercise to exhaustion test was; (iii) a statistically significant accumulation of blood lactate clearance after 10 min of myofascial tissue rolling with a large effect size. Furthermore, rolling 15 out of 30 min of recovery between the two high intensity tests gave; (iv) a non-significant trivial effect on average power, but (v) a tendency for a medium positive effect on peak power (*p* = 0.084; *d* = 0.7) in the consecutive 30 s Wingate test.

### 4.1. The Effect of Pre Exercise Myofascial Tissue Release on Performance to Exhaustion

In this study, it was hypothesized that quadriceps myofascial tissue release could potentially affect endurance performance through reducing local limitations of aerobic power and/or anaerobic capacity. The aerobic performance indicator, VO_2peak_, has been shown to be both centrally and locally limited when highly trained cross-country skiers (VO_2peak_ > 65 mL·kg^-1^·min^−1^) were tested in different exercise modes [44]. However, in the present study, quadriceps myofascial tissue release gave a trivial effect with no statistically significant difference on VO_2peak_, which is in line with previous studies [18,45].

On the other hand, blood lactate concentration was statistically significantly higher after the test to exhaustion on the day with rolling (Table 1). Hence, indicating a larger buffering of blood lactate during the test shown by the nearly equivalent blood lactate concentration after the 1st warm up (0.33 ± 0.68 mmol·L^−1^; Table 2) and the respiratory exchange ratio observed in both testing days (Table 1). Theoretically, an increase of 1 mmol·L^−1^ in blood lactate concentration is equivalent to ~3 mL·kg^−1^ of oxygen consumed with a respiratory exchange ratio = 1.0 [46]. Thus, theoretically the average person in the present study could increase total energy expenditure equivalent to energy from approximately 335 mL of oxygen. Such an increase in anaerobic capacity would be expected to enhance performance in terms of time to exhaustion (at an effort equal to VO_2peak_, the 335 mL of extra oxygen would last ~6 s). Interestingly, examining time to exhaustion indicate that participants continued on average 6.1 (9.9) s longer on the day with rolling compared to the day without rolling (Table 2). Nevertheless, the change was not statistically notable, and the effect size of the myofascial tissue rolling condition was found to be small (*d* = 0.219; Table 1).

### 4.2. The Myofascial Tissue Release Effect on Recovery and Consecutive Test of Anaerobic Capacity

In this study, a 30 min recovery between the two performance tests was used as it simulates a sport specific condition [26]. Considering the intensity and duration of the speed skating races, studies from other sports indicate that 30 min of recovery might be close to enough between races [2,7]. However, in the present study, the skaters still had elevated blood lactate concentration at the end of the second warm-up compared to the first, indicating the importance of recovery strategy to performance.

The rate of blood lactate clearance tended to be largest during the early phase of recovery and the results showed a statistically significant larger accumulated blood lactate clearance (with a large effect size) after myofascial tissue rolling for 10 min (Table 2). However, the difference in the accumulated blood lactate clearance observed could be due to: (i) the effect of myofascial tissue release on test to exhaustion performance (higher tolerance to high intensity exercise indicated by higher blood lactate concentration and longer working time); (ii) due to myofascial tissue release during the recovery time provided. Nevertheless, maintaining the myofascial tissue release from 10th to 15th min during recovery did not increase the rate of blood lactate clearance. On the other hand, the average difference in blood lactate clearance decreased between the 15th and the 20th min (Table 2).

There is no doubt that the test to exhaustion was highly intensive. So was the 30 s Wingate test, which was used as the second performance test. It estimates anaerobic capacity [41] and changes in the Wingate performance test have been shown to predict changes in 1500 m speed skating performance [29]. A time gain of ~0.3 s on 1500 m is a worthwhile change for a world-class skater (>10% chance for a medal candidate), while the smallest worthwhile change is somewhat larger for junior skaters with larger variations [47]. Hofman et al. [29] reported the smallest worthwhile change in Wingate peak power and mean power to be 0.38 and 0.14 W/kg for females, and 0.29 and 0.12 W/kg for males, respectively. The results from the present study showed a tendency towards a medium positive effect on peak power on the day with myofascial tissue release (Table 3). However, the peak power and mean power differences observed in the present study were smaller than the smallest worthwhile changes reported by Hofman et al. [29].

### 4.3. Methodological Considerations

Contrary effects of myofascial tissue release have been reported in the literature [5,21,22,23,25,26,27,30] using a different number of participants, release techniques, and equipment. The major challenges in this study were the low number of participants (8 participants), duration of the treatment (acute effect), and the interval (5 min) in which the blood lactate concentration was measured. The sample size in the present study was small for detecting the effects of myofascial tissue release. However, it is challenging to find a large homogeneous sample size from individual elite sports that share the same training routines. Furthermore, data from small samples could still be of interest for coaches and athletes and could be the basis for future meta-analysis studies (i.e., [10]). In this respect, note that males performed better than females on VO_2peak_ and power. Hence, SD between all participants is large for these variables, while within subject differences between testing days did not indicate that myofascial tissue release to affect gender differently. Further, blood lactate concentration and respiratory exchange ratio were not affected by gender due to the fact that blood lactate concentration and the respiratory exchange ratio are exercise intensity and duration dependent [10,11,12].

## 5. Conclusions

Despite indications for potential benefit (of the quadriceps myofascial tissue release using a mechanical self-myofascial release roller-Massager) on blood lactate concentration and Wingate peak-power, the acute myofascial tissue release gave no marked performance improvements, nor indications of negative effects. The results from this study and past studies suggest the need for examining the chronic effect of self-induced myofascial tissue release on performance and recovery. Furthermore, integrating a measure of participants subjective experience before and after the myofascial tissue release would be of great interest and might provide more room for possible explanations.

## Figures and Tables

**Table 1:** Measures of the ramp cycle ergometer test to exhaustion.

					95% CI for Mean Difference		
Variables	TT (SD)	CT (SD)	Mean Difference (SE)	*p*-Value	Lower	Upper	Cohen’s *d*
Time to exhaustion (s)	441 (106)	435 (93)	6.1 (9.9)	0.555	−17.2	29.5	0.219
VO_2Peak_ (mL/kg)	54.9 (9.2)	55.2 (9.1)	−0.34 (0.71)	0.650	−2.02	1.35	−0.168
RER	1.17 (0.03)	1.16 (0.05)	0.01 (0.01)	0.462	−0.02	0.05	0.275
Blood lactate concentration at exhaustion	16.6 (3.9)	14.8 (4.1)	1.75 (0.73) *	0.047	0.03	3.47	0.849

Note. * = *p* < 0.05; TT = treatment test; CT = control test; SD = standard deviation; SE = standard error of the difference; CI = confidence interval; RER = respiratory exchange ratio.

**Table 2:** Measures of blood lactate concentration and clearance on a five min interval up to 20 min.

					95% CI for Mean Difference		
Measuring Time-Point	TT (SD)	CT (SD)	Mean Difference (SE)	*p*-Value	Lower	Upper	Cohen’s *d*
At end of 1st warm-up	2.4 (1.2)	2.0 (0.8)	0.33 (0.24)	0.217	−0.24	0.89	0.480
At exhaustion	16.6 (3.9)	14.8 (4.1)	1.75 (0.73) *	0.047	0.03	3.47	0.849
At 5 min	3.3 (1.9)	2.6 (2.0)	0.76 (0.74)	0.338	−0.99	2.52	0.363
At 10 min	5.3 (1.5)	4.2 (1.6)	1.10 (0.27) *	0.004	0.47	1.73	1.466
At 15 min	7.1 (2.2)	5.9 (1.8)	1.16 (0.82)	0.197	−0.77	3.09	0.504
At 20 min	8.6 (1.8)	7.8 (1.6)	0.79 (0.74)	0.320	−0.95	2.53	0.378
At end of 2nd warm-up	4.0 (3.2)	3.2 (2.4)	0.89 (0.52)	0.128	−0.33	2.10	0.610

Note. * = *p* < 0.05; TT = treatment test; CT = control test; SD = standard deviation; SE = standard error of the difference; CI = confidence interval.

**Table 3:** Measures of Wingate anaerobic cycle ergometer test.

					95% CI for Mean Difference		
Wingate Measures	TT (SD)	CT (SD)	Mean Difference (SE)	*p*-Value	Lower	Upper	Cohen’s d
PP (watt)	735 (159)	718 (152)	16.6 (8.2)	0.084	−2.93	36.05	0.710
RPP (watt/kg)	11.4 (1.5)	11.2 (1.4)	0.24 (0.13)	0.109	−0.07	0.54	0.649
AP (watt)	629 (144)	630 (140)	−1.5 (6.8)	0.832	−17.48	14.51	−0.078
RoF (%)	26.3 (6.8)	24.3 (7.9)	2.0 (1.5)	0.218	−1.49	5.47	0.478

Note. TT = treatment test; CT = control test; SD = standard deviation; SE = standard error of the difference; CI = confidence interval; PP = peak power; RPP = relative peak power; AP = average power; RoF = rate of fatigue.

## References

[1] Bogdanis G.C., Nevill M.E., Boobis L.H., Lakomy H.K., Nevill A.M. (1995). Recovery of power output and muscle metabolites following 30 s of maximal sprint cycling in man. J. Physiol..

[2] Losnegard T., Andersen M., Spencer M., Hallén J. (2015). Effects of active versus passive recovery in sprint cross-country skiing. Int. J. Sports Physiol. Perform..

[3] Monedero J., Donne B. (2000). Effect of recovery interventions on lactate removal and subsequent performance. Int. J. Sports Med..

[4] Herda T.J., Cramer J.T., Haff G.G., Triplett N.T. (2016). Bioenergetics of Exercise and Training, in Essentials of Strength Training and Conditioning.

[5] Cairns S.P. (2006). Lactic acid and exercise performance: Culprit or friend?. Sports Med..

[6] Kraemer W.J., Marchitelli L., Gordon S.E., Harman E., Dziados J.E., Mello R., Frykman P., McCurry D., Fleck S.J. (1990). Hormonal and growth factor responses to heavy resistance exercise protocols. J. Appl. Physiol. (1985).

[7] D’Amico A., Paolone V. (2017). The Effect of Foam Rolling on Recovery Between two Eight Hundred Metre Runs. J. Hum. Kinet..

[8] Poppendieck W., Wegmann M., Ferrauti A., Kellmann M., Pfeiffer M., Meyer T. (2016). Massage and Performance Recovery: A Meta-Analytical Review. Sports Med..

[9] Menzies P., Menzies C., McIntyre L., Paterson P., Wilson J., Kemi O.J. (2010). Blood lactate clearance during active recovery after an intense running bout depends on the intensity of the active recovery. J. Sports Sci..

[10] Hohenauer E., Taeymans J., Baeyens J.P., Clarys P., Clijsen R. (2015). The Effect of Post-Exercise Cryotherapy on Recovery Characteristics: A Systematic Review and Meta-Analysis. PLoS ONE.

[11] Versey N.G., Halson S.L., Dawson B.T. (2013). Water immersion recovery for athletes: Effect on exercise performance and practical recommendations. Sports Med..

[12] Branco B.H.M., Fukuda D.H., Andreato L.V., da Silva Santos J.F., Esteves J.V.D.C., Franchini E. (2016). The Effects of Hyperbaric Oxygen Therapy on Post-Training Recovery in Jiu-Jitsu Athletes. PLoS ONE.

[13] Lundberg T.R., Howatson G. (2018). Analgesic and anti-inflammatory drugs in sports: Implications for exercise performance and training adaptations. Scand. J. Med. Sci. Sports.

[14] Brown F., Gissane C., Howatson G., Van Someren K., Pedlar C., Hill J. (2017). Compression Garments and Recovery from Exercise: A Meta-Analysis. Sports Med..

[15] Sands W.A., McNeal J.R., Murray S.R., Ramsey M.W., Sato K., Mizuguchi S., Stone M.H. (2013). Stretching and Its Effects on Recovery: A Review. Strength Cond. J..

[16] D’Amico A.P., Gillis J. (2017). The influence of foam rolling on recovery from exercise-induced muscle damage. J. Strength Cond. Res..

[17] Rey E., Padrón-Cabo A., Costa P.B., Barcala-Furelos R. (2017). The Effects of Foam Rolling as a Recovery Tool in Professional Soccer Players. J. Strength Cond. Res..

[18] Cheatham S.W., Kolber M.J., Cain M., Lee M. (2015). The Effects Of Self-Myofascial Release Using A Foam Roll Or Roller Massager On Joint Range Of Motion, Muscle Recovery, And Performance: A Systematic Review. Int. J. Sports Phys. Ther..

[19] Behara B., Jacobson B.H. (2017). Acute Effects of Deep Tissue Foam Rolling and Dynamic Stretching on Muscular Strength, Power, and Flexibility in Division I Linemen. J. Strength Cond. Res..

[20] Bradbury-Squires D.J., Noftall J.C., Sullivan K.M., Behm D.G., Power K.E., Button D.C. (2015). Roller-massager application to the quadriceps and knee-joint range of motion and neuromuscular efficiency during a lunge. J. Athl. Train..

[21] Macdonald G.Z., Button D.C., Drinkwater E.J., Behm D.G. (2014). Foam rolling as a recovery tool after an intense bout of physical activity. Med. Sci. Sports Exerc..

[22] Healey K.C., Hatfield D.L., Blanpied P., Dorfman L.R., Riebe D. (2014). The effects of myofascial release with foam rolling on performance. J. Strength Cond. Res..

[23] Park D.J., Hwang Y.I. (2016). A pilot study of balance performance benefit of myofascial release, with a tennis ball, in chronic stroke patients. J. Bodyw. Mov. Ther..

[24] Mikesky A.E., Bahamonde R.E., Stanton K., Alvey T., Fitton T. (2002). Acute effects of The Stick on strength, power, and flexibility. J. Strength Cond. Res..

[25] Sullivan K.M., Silvey D.B., Button D.C., Behm D.G. (2013). Roller-massager application to the hamstrings increases sit-and-reach range of motion within five to ten seconds without performance impairments. Int. J. Sports Phys. Ther..

[26] International Skating Union (2018). Special Regulations & Technical Rules—Speed Skating.

[27] Foster C., Rundell K.W., Snyder A.C., Stray-Gundersen J., Kemkers G., Thometz N., Broker J., Knapp E. (1999). Evidence for restricted muscle blood flow during speed skating. Med. Sci. Sports Exerc..

[28] Rundell K.W. (1996). Compromised oxygen uptake in speed skaters during treadmill in-line skating. Med. Sci. Sports Exerc..

[29] Hofman N., Orie J., Hoozemans M.J., Foster C., de Koning J.J. (2017). Wingate Test as a Strong Predictor of 1500-m Performance in Elite Speed Skaters. Int. J. Sports Physiol. Perform..

[30] De Koning J.J., Foster C., Lampen J., Hettinga F., Bobbert M.F. (2005). Experimental evaluation of the power balance model of speed skating. J. Appl. Physiol. (1985).

[31] Borg G.A. (1962). Physical Performance and Perceived Exertion.

[32] Vyaire M. (2016). Vyntus CPX Powered by SentrySuite. https://www.vyaire.com/Documents/international/brochures/respiratory-care/cardiopulmonary/RC_Vyntus-CPX_BR_EN.pdf.

[33] Garzon M., Gayda M., Nigam A., Comtois A.S., Juneau M. (2017). Immersible ergocycle prescription as a function of relative exercise intensity. J. Sport Health Sci..

[34] Gibson A.L., Wagner D.R., Heyward V.H. (2019). Advanced Fitness Assessment and Exercise Prescription.

[35] Edvardsen E., Hem E., Anderssen S.A. (2014). End criteria for reaching maximal oxygen uptake must be strict and adjusted to sex and age: A cross-sectional study. PLoS ONE.

[36] Fletcher I.M. (2010). The effects of precompetition massage on the kinematic parameters of 20-m sprint performance. J. Strength Cond. Res..

[37] Moran R.N., Hauth J.M., Rabena R. (2018). The effect of massage on acceleration and sprint performance in track & field athletes. Complement. Ther. Clin. Pract..

[38] Brummitt J. (2008). The role of massage in sports performance and rehabilitation: Current evidence and future direction. N. Am. J. Sports Phys. Ther. NAJSPT.

[39] Hinds T., McEwan I., Perkes J., Dawson E., Ball D., George K. (2004). Effects of massage on limb and skin blood flow after quadriceps exercise. Med. Sci. Sports Exerc..

[40] MacIntosh B.R., Rishaug P., Svedahl K. (2003). Assessment of peak power and short-term work capacity. Eur. J. Appl. Physiol..

[41] Bar-Or O. (1987). The Wingate anaerobic test. An update on methodology, reliability and validity. Sports Med..

[42] Shalfawi S.A.I. (2016). Statistical Use in Applied Sport Research: Methodological and Ethical Challenges. Strength Cond. J..

[43] Cohen J. (1988). Statistical Power Analysis for the Behavioral Sciences.

[44] Losnegard T., Schäfer D., Hallén J. (2014). Exercise economy in skiing and running. Front. Physiol..

[45] Peacock C.A., Krein D.D., Antonio J., Sanders G.J., Silver T.A., Colas M. (2015). Comparing Acute Bouts of Sagittal Plane Progression Foam Rolling vs. Frontal Plane Progression Foam Rolling. J. Strength Cond. Res..

[46] di Prampero P.E., Ferretti G. (1999). The energetics of anaerobic muscle metabolism: A reappraisal of older and recent concepts. Respir. Physiol..

[47] Noordhof D.A., Mulder R.C., de Koning J.J., Hopkins W.G. (2016). Race Factors Affecting Performance Times in Elite Long-Track Speed Skating. Int. J. Sports Physiol. Perform..

